# Diversity analysis of sea anemone peptide toxins in different tissues of *Heteractis crispa* based on transcriptomics

**DOI:** 10.1038/s41598-024-58402-2

**Published:** 2024-04-01

**Authors:** Qiqi Guo, Jinxing Fu, Lin Yuan, Yanling Liao, Ming Li, Xinzhong Li, Bo Yi, Junqing Zhang, Bingmiao Gao

**Affiliations:** 1 Engineering Research Center of Tropical Medicine Innovation and Transformation, Ministry of Education, International Joint Research Center of Human-machine Intelligent Collaborative for Tumor Precision Diagnosis and Treatment of Hainan Province, School of Pharmacy, Hainan Medical University, Haikou, China; 2 School of Health and Life Sciences, Teesside University, Middlesbrough, UK; 3 Department of Pharmacy, 928th Hospital of PLA Joint Logistics Support Force, Haikou, China

**Keywords:** Molecular biology, Ocean sciences

## Abstract

Peptide toxins found in sea anemones venom have diverse properties that make them important research subjects in the fields of pharmacology, neuroscience and biotechnology. This study used high-throughput sequencing technology to systematically analyze the venom components of the tentacles, column, and mesenterial filaments of sea anemone *Heteractis crispa*, revealing the diversity and complexity of sea anemone toxins in different tissues. A total of 1049 transcripts were identified and categorized into 60 families, of which 91.0% were proteins and 9.0% were peptides. Of those 1049 transcripts, 416, 291, and 307 putative proteins and peptide precursors were identified from tentacles, column, and mesenterial filaments respectively, while 428 were identified when the datasets were combined. Of these putative toxin sequences, 42 were detected in all three tissues, including 33 proteins and 9 peptides, with the majority of peptides being ShKT domain, β-defensin, and Kunitz-type. In addition, this study applied bioinformatics approaches to predict the family classification, 3D structures, and functional annotation of these representative peptides, as well as the evolutionary relationships between peptides, laying the foundation for the next step of peptide pharmacological activity research.

## Introduction

Sea anemones (Cnidaria: Anthozoa: Hexacorallia: Actiniaria) are an order of marine animals found in deep oceans and shallow coastal regions around the world, including two suborders: Anenthemonae and Enthemonae^1^. Cnidarians are one of the oldest venomous lineages in existence. Molecular and fossil evidence suggesting that they appeared more than 750 million years ago, before the Ediacaran period^2–4^. Like other cnidarians, sea anemones store venom in specialized cells known as nematocysts, which have venom-filled capsules and inverted tubules^5,6^. Contact with prey causes an explosive eversion of the tubule, piercing the target organism and releasing venom for predation, defense, or competitive deterrence^7^. The endodermal and ectodermal gland tissue of sea anemones contains venom, revealing an alternative venom-delivery mechanism in sea anemones^8,9^.

Sea anemone venom contains complex proteinaceous (peptides and proteins) and non-proteinaceous components (e.g., quaternary ammonium compounds, purines, and biogenic amines)^10^. Sea anemone toxins disrupt many targets, including voltage-gated sodium (Nav) and potassium (Kv) channels, acid-sensing ion channels (ASIC), transient receptor potential vanilloid 1 (TRPV1), and transient receptor potential ankyrin 1 (TRPA1)^11–15^. Many peptide toxins in sea anemones have been studied for their potential as pharmaceutical tools or treatments. The sea anemone venom protein ShK of *Stichodactyla helianthus* inhibited Kv1.3 with an IC50 in the low picomolar range^16,17^. The efficacy of ShK and its homologs in the treatment of human autoimmune disorders, including rheumatoid arthritis, multiple sclerosis, and type I diabetes, have been demonstrated in animal models^18–20^. Moreover, the phase I trials of Dalazatide (formerly ShK-186) in the treatment of psoriasis have been successfully concluded^21^.

Sea anemone *Heteractis crispa (H. crispa),* also known as the leathery sea anemone, long tentacle anemone or purple tip anemone, belongs to Heteractidae family and is native to the Indo-Pacific region^22–24^. In 1994, Mebs mentioned the sea anemone *Heteractis crispa*^25^, which has the valid name *Radianthus crispa* on the WoRMS website. In 2010, Fedorov *et al*. published an article in which they mentioned that *Radianthus macrodactylus* is equivalent to *Heteractis crispa*^26^. Some toxins have been detected in *H. crispa*, mainly actinoporin, Kunitz-type protease inhibitors, Nav channel toxins, and Kv channel toxins^26–29^, and the venom assembly in the tentacles, mesentery filaments, and columns of three species of sea anemones (*Anemonia sulcata*, *H. crispa*, and *Megalactis griffithsi*) has been investigated, and the number of toxin-like genes varies significantly between tissues and species^22,30,31^. The high-throughput sequencing (HTS) technology has been increasingly applied in studying sea anemone venom components, such as *Stichodactyla haddoni* and *Anthopleura dowii*^32–34^. In this study, we applied HTS technology to identify the protein and peptide components in *H. crispa* and compared the distribution of peptide toxins in different tissues, this lays the foundation for in-depth research on *H. crispa* peptide toxins and provides new potentials for marine drug development.

## Results

### Transcriptome sequence assembly

We applied the BUSCO suite tools to assess the completeness of transcriptomes^35^. We found that overall, the BUSCO match values were within the expected range for both a complete single copy and duplicated copies of BUSCO (S/D). Whether in the Tentacles, Column, Mesenterial filaments, or their combination (Combine) dataset, there were a large number of Complete BUSCOs (C) accounting for more than 90% of the Total BUSCO groups, while the proportion of Fragmented BUSCOs (F) and Missing BUSCOs (M) in the Total BUSCO groups was extremely low (Table S1).

The sequence and assembly of *H. crispa* transcriptome were generated and then submitted to the National Center for Biotechnology Information (NCBI) (BioProject: PRJNA893400, and SRA accession: SRX17999840, SRX17999841, and SRX17999842). The total Reads of the Tentacles (83,799,076), Column (69,427,990), and Mesenterial filaments (67,486,092) were merged into one combined dataset (Combine). After filtering out low-quality reads, about 81.9 million (81,961,116), 67.8 million (67,828,874), 66.2 million (66,235,446), and 120 million reads were obtained from the Tentacles, Column, Mesenterial filaments, and the Combine dataset, respectively. The HTS data was assembled into transcript sequences by using Inchworm, Chrysalis, and Butterfly assembly tools^36,37^ which generated 288,563 contigs with a mean length of 1,001 bp and an N50 length of 1,829 bp. Meanwhile, 183,198 non-redundant unigenes with a mean length of 769 bp and an N50 length of 1,199 bp were obtained by splicing and removing redundant sequences (Table S2).

Annotation was performed based on five databases to examine unigene functions: Nr (NCBI non-redundant protein sequence), KOG (Eukaryotic Orthologous Groups), Uniprot (Universal Protein), GO (Gene Ontology), and KEGG (Kyoto Encyclopedia of Genes and Genomes). A total of 183,198 unigenes were grouped into these databases: Nr (37,932 unigenes), Uniprot (40,920 unigenes), GO (29,531 unigenes), KEGG (16,167 unigenes), and KOG (20,259 unigenes) respectively, while there were 139,585 unigenes lacking annotation in these databases (Figure S1). Moreover, 19,414 unigenes were enriched into 33 KEGG pathways and assigned into five primary categories: processing environmental information (2,799, 14.42%), cellular processes (2,801, 14.42%), genetic information processing (2,539, 13.08%), metabolism (6,221, 32.04%), and organismal systems (5,054, 26.03%). Most of these unigenes were assigned to metabolism, and the global and overview maps of metabolism contained the most annotated unigenes (Figure S2). 18,016 unigenes were annotated in KOG and categorized into 25 different molecular families (Figure S3)."General function cluster prediction only" (3,831 unigenes, 21.26%) was the largest of these KOG categories, followed by "Signal transduction pathways" (2,419 unigenes, 13.43%), "Posttranslational modification, protein turnover, chaperones" (1,893 unigenes, 10.51%), and "Nuclear structure" (10 unigenes, 0.06%). GO analysis demonstrated that 110,279 unigenes were categorized into three categories: Cellular Component (27,969, 25.36%), Biological Process (46,361, 42.13%), and Molecular Function (35,849, 32.51%), which were further classified into 49 functional groups. The top four enriched functional groups were binding (14,521 unigenes), cellular process (14,127 unigenes), catalytic activity (13,471 unigenes), and metabolic function (13,024 unigenes) (Figure S4).

**Figure 1:**
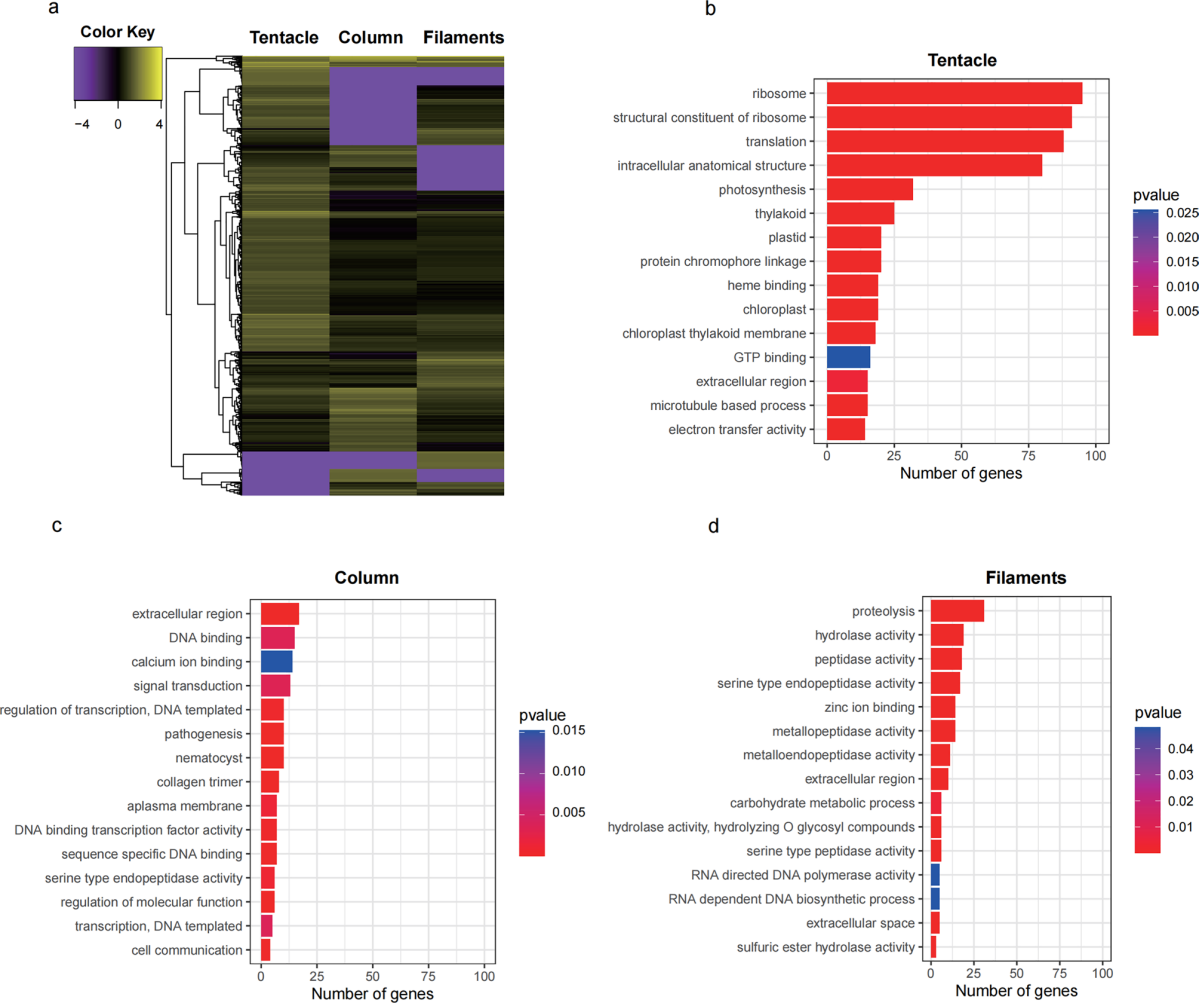
Cluster heatmap and genes annotated by GO analysis in three different tissues. (**a**) Cluster heatmap of three different tissues. (**b**) Bar chart of the number of genes in tentacles. (**c**) Bar chart of the number of genes in the column. (**d**) Bar chart of the number of genes in the mesenterial filaments.

Hierarchical clustering analysis demonstrated that the Tentacles, Column, and Mesenterial filaments in *H. crispa* were well distinguished, and all gene sequences were divided into three categories (Fig. 1a). GO analysis of gene expression indicated (Fig. 1b–d) that the number of genes expressed in Tentacles was greater than the number of genes expressed in the Column and Mesenterial filaments. In Tentacles, ribosomes, structural constitutions of ribosomes, translation, and internal anatomical structure have the largest number of genes. Meanwhile, the extracellular region and proteolysis have the largest gene numbers in the Column and Mesenterial filaments.

### Family classification of proteins and peptide toxins in the *H. crispa* transcriptome

**Figure 2:**
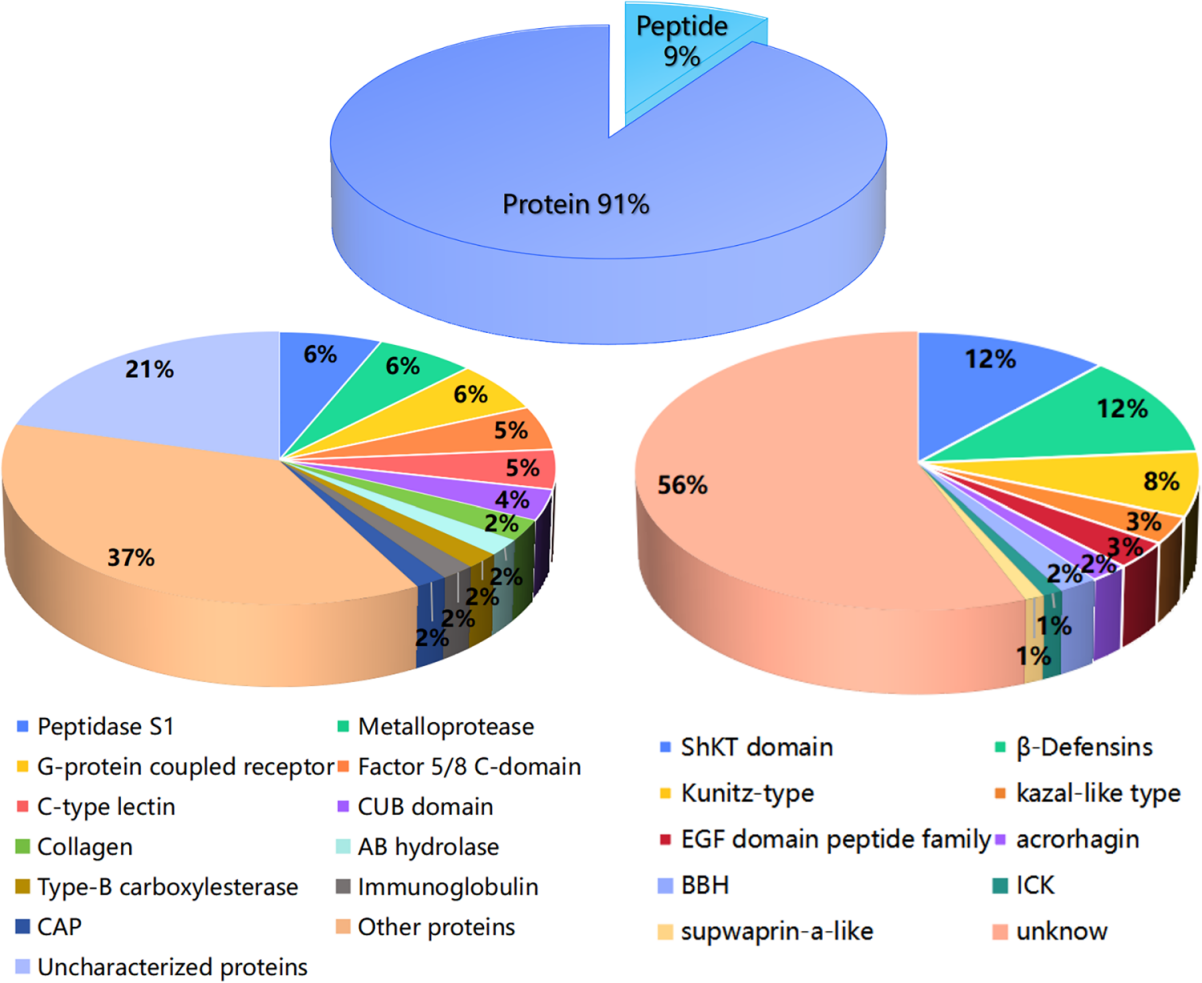
Families of putative protein and peptide toxins in *H. crispa* transcriptome. Based on their amino acid sequences and cysteine scaffolds, the 956 protein sequences and 93 peptide sequences with significant BLAST hits to manually curated lists of animal toxins in UniProt (www.uniprot.org/program/Toxins) were assigned to distinct toxin families.

A total of 1049 transcripts were assessed and categorized into 60 families regarding predicted functions, which were assessed according to their amino acid sequence identity, of which 91.0% were proteins and 9.0% were peptides (amino acids ≤ 80) (Fig. 2, Table S3). Most protein components matched Peptidase S1, metalloprotease, G-protein coupled receptor, and Factor 5/8 C-domain. The important and well-known actinoporins family was included in the above statement (Figures S5,6). Moreover, the peptide components were related to the ShKT domain, β-defensin, and Kunitz-type.

### Comparative analysis of protein and peptide toxins in different tissues of *H. crispa*

**Figure 3:**
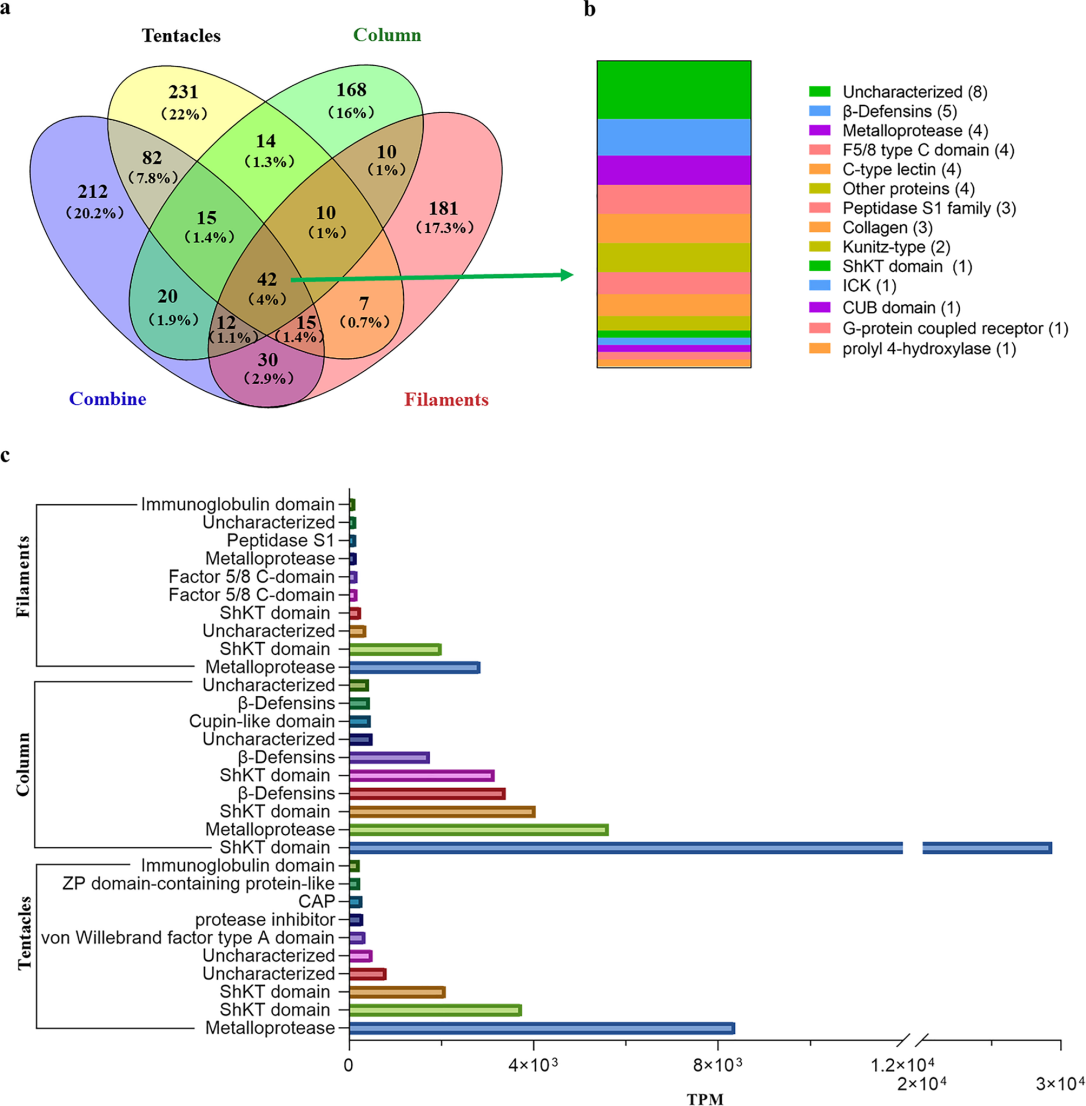
Transcripts of protein and peptide toxins from several *H. crispa* tissues are compared. (**a**) Correlation between datasets of putative protein and neurotoxic peptide detected from *H. crispa* combine, tentacles, column, and mesenterial filaments. (**b**) 42 putative protein and peptide transcripts from various *H. crispa* tissues. (**c**) The ten most greatly expressed protein and peptide transcripts from different *H. crispa* tissues.

A total of 416, 291, 307, and 428 putative proteins and peptide toxins precursors were detected from the tentacles, column, mesenterial filaments, and their combined dataset, respectively. Figure 3a depicts the comparative distributions of protein and peptide toxins. Of these putative proteins and toxic precursors, 42 were common in these four datasets, of which 33 were proteins and 9 were peptides. A total of 81 were shared by tentacles and column, 74 were shared by tentacles and mesenterial filaments, and 74 were common in both column and mesenterial filaments. These 42 common toxins precursors across four databases were classified into 14 families: β-defensin, metalloproteinase, Kunitz-type, and ShKT domain (Fig. 3b). For each protein and peptide toxin, transcripts Per Kilobase of exon model per Million mapped reads (TPM) values were calculated representing transcription levels. The top ten protein and peptide toxins (with the highest TPM values) in each dataset were assigned. The metalloprotease and ShKT domains derived from the tentacles were expressed at high levels, while the ShKT domain, metalloprotease, and β-defensin derived from the column were expressed at high levels too. However, various proteins and peptide toxins derived from mesenterial filaments were downregulated, among which metalloprotease and ShKT domains were still the highest (Fig. 3c). Therefore, protein and peptide toxins in the ShKT domain and metalloprotease were highly expressed in three *H. crispa* tissues. Additionally, the ShKT domain included protein and peptide toxins with the highest expressions in the column. Surprisingly, only β-defensin-like peptides were highly expressed in the column but not in the other two tissues, which deserves further studies.

### Cysteine pattern analysis of sea anemone peptide toxins

**Figure 4:**
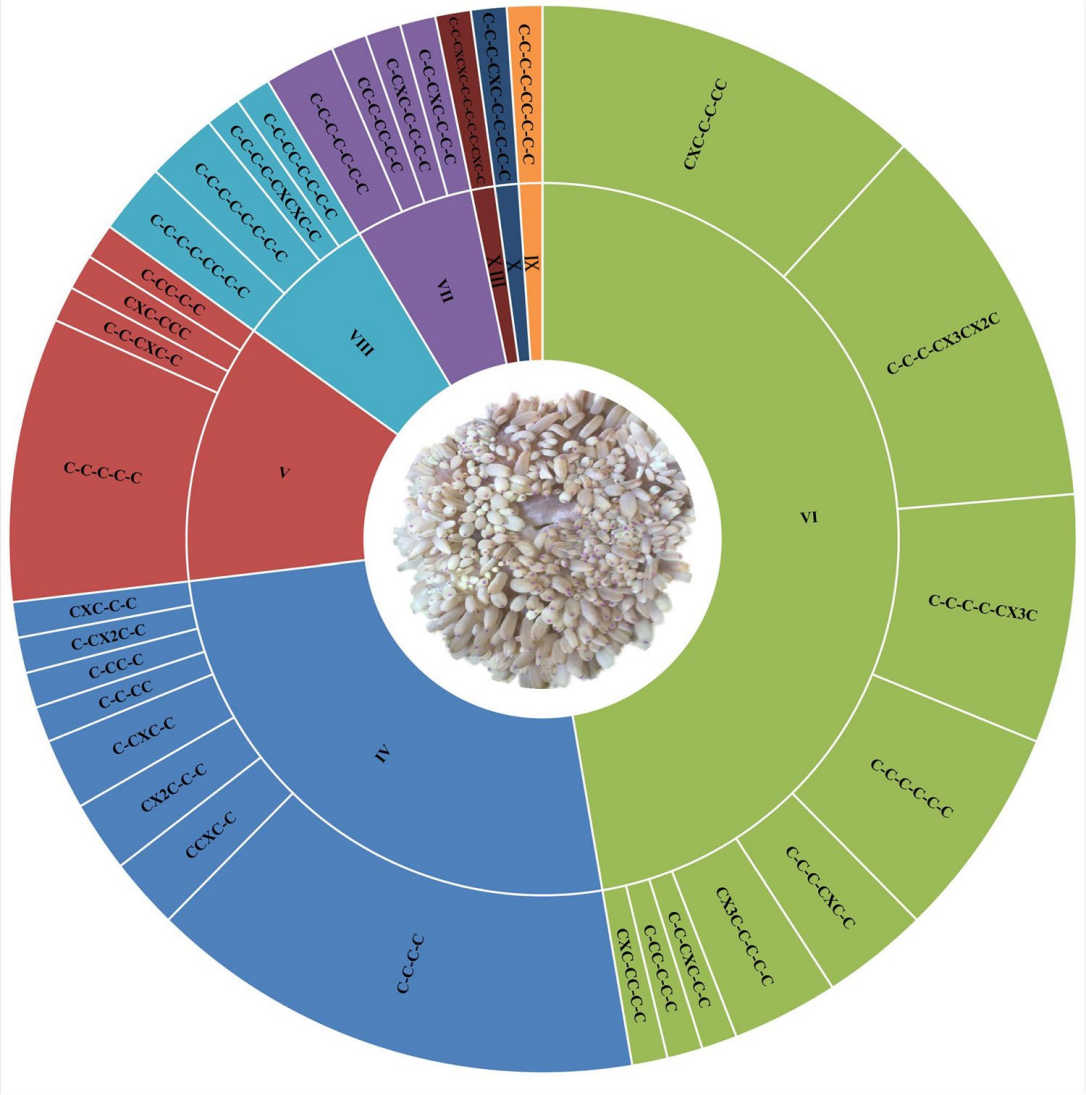
Classification of peptide toxic cysteine patterns from *H. crispa*.

The nomenclature and classification of cysteine patterns in sea anemone neurotoxic peptides have been reported by Kozlov^38^ and Gao et al^39^. In this study, a total of 93 peptide toxins were obtained from the tentacles, column, mesenterial filaments, and combined datasets and named Hc-01~Hc-93 in order (Table S4). Many cysteines exist in sea anemone peptide toxins, and cysteine structural scaffolds are diverse. According to our previously proposed classification method^39,40^, cysteine patterns of these 93 peptides were split into eight broad categories and several subcategories (Fig. 4). The most common peptide structures have six cysteines and three disulfide bond patterns (VI), accounting for 47.31% followed by those having four cysteines producing two disulfide bond patterns (IV), accounting for 25.81%. Furthermore, although most peptides possess an even number of cysteines, there is a small proportion of peptides characterized by an odd number of cysteine residues. The peptide toxins of IV-type and VI-type in sea anemones may be engaged in capturing prey, defending against predators, or repulsing competitors, indicating that these peptide toxins have rich targeting activities^41,42^. These peptide toxins have potential biotechnological applications and provide rich resources for the development of new drugs.

### Sequence analysis of typical sea anemone peptide toxins

**Figure 5:**
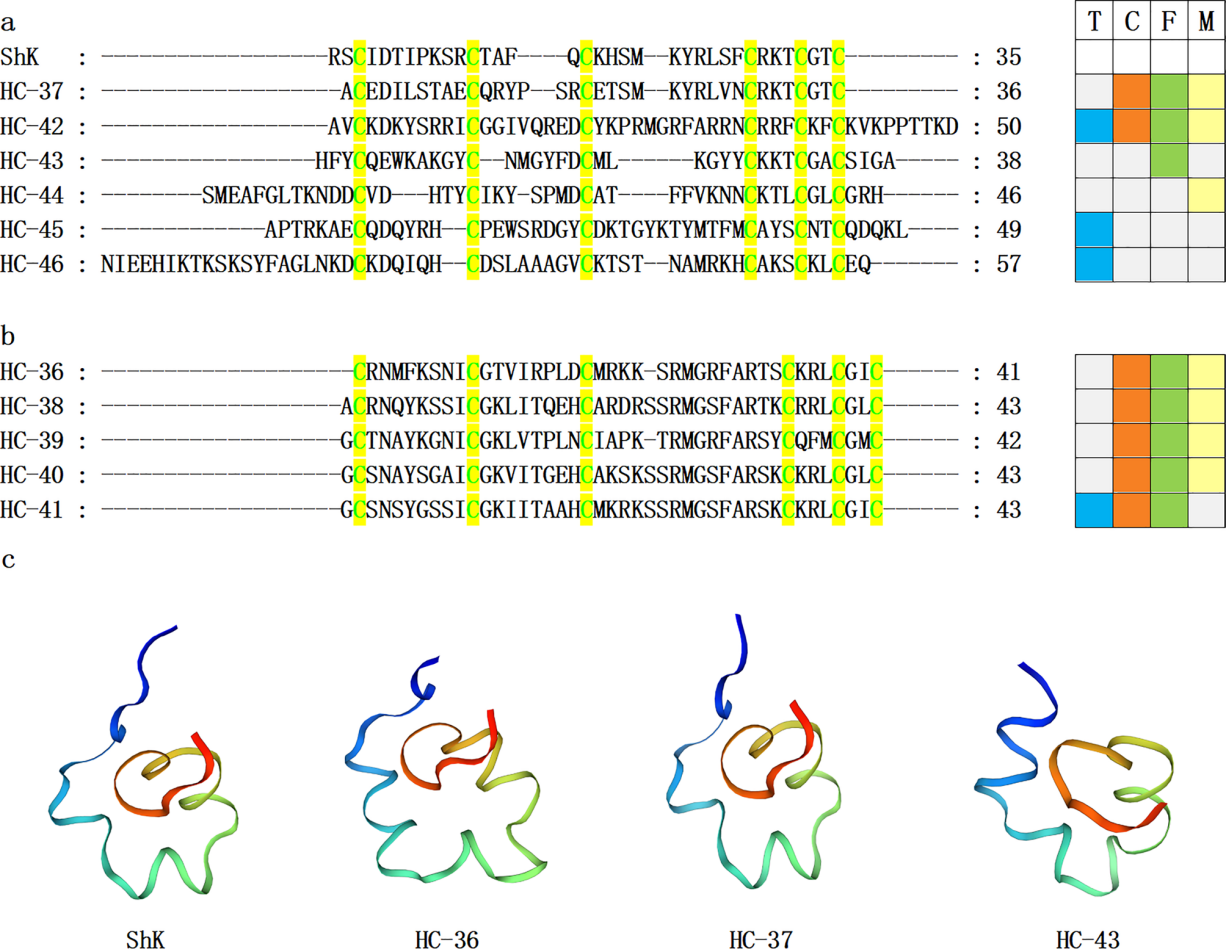
Sea anemone ShKT domain mature peptide sequences. (**a–b**) The conserved cysteine residues are highlighted with green text on yellow background. T, C, F, and M respectively represent tentacles, column, mesenterial filaments, and combine, highlighted in blue, orange, green, and yellow. (**c**) Homology modeling and ShK prediction of sea anemone mature peptides HC-36, HC-37, and HC-43 (PDB 4LFQ).

**Figure 6:**
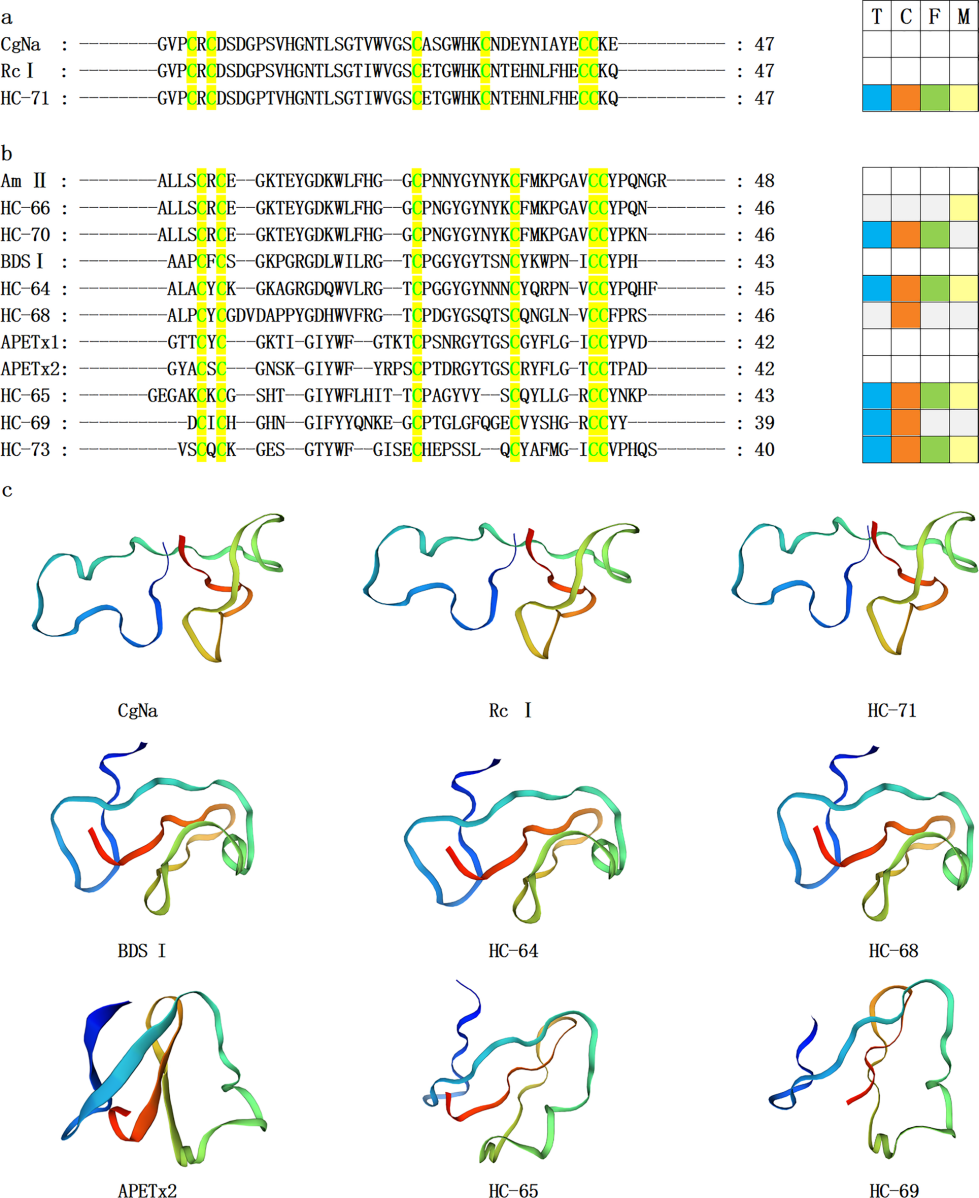
β-defensin-like sea anemone mature peptide sequences. (**a–b**) The conserved cysteine residues are highlighted with green text on yellow background. T, C, F, and M respectively represent tentacles, column, mesenterial filaments, and combine, highlighted in blue, orange, green, and yellow. (**c**) Homology modeling prediction of several mature peptides from sea anemones with CgNa (PDB 2H9X), BDS I (PDB 1BDS), and APETx2 (PDB 2MUB).

**Figure 7:**
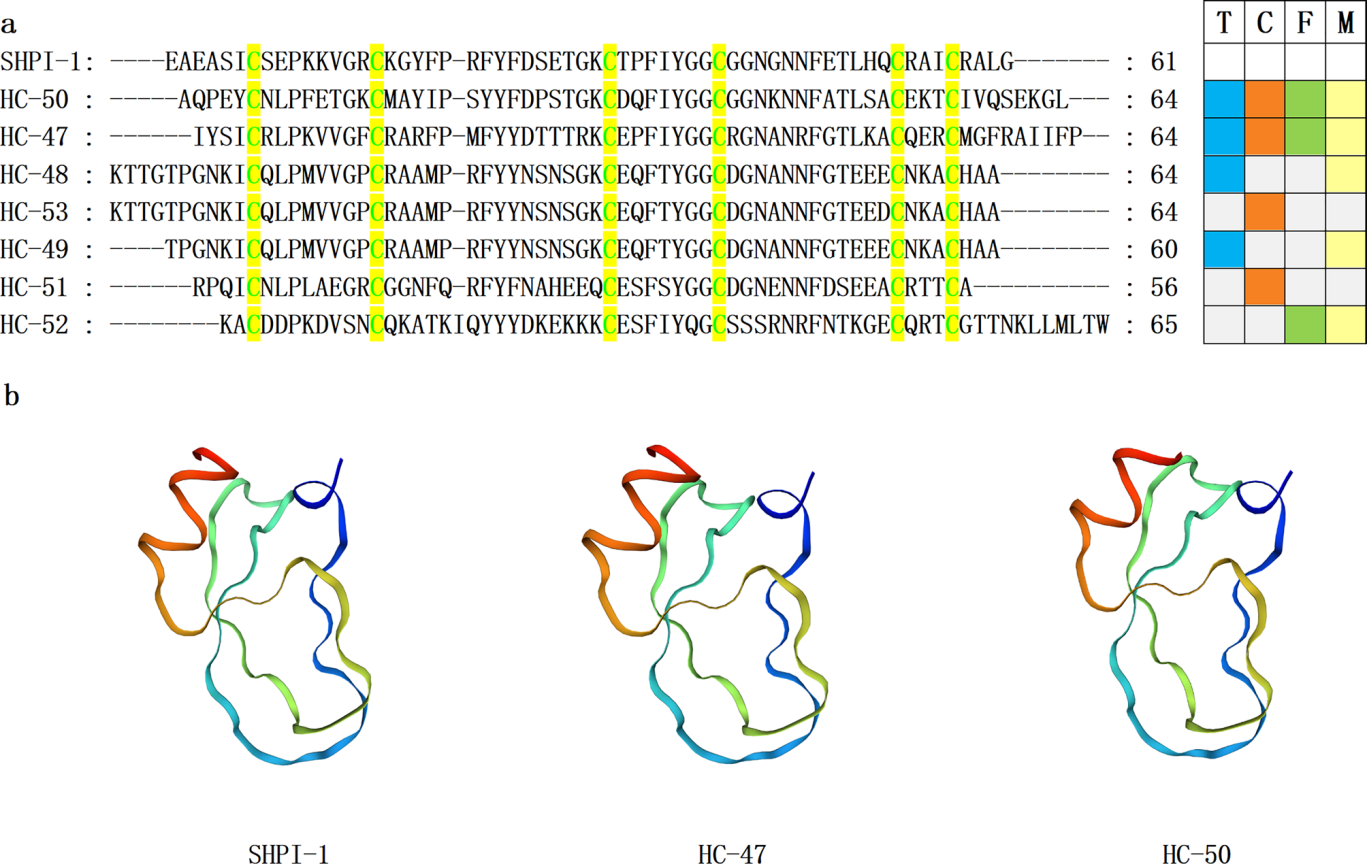
Representative mature peptide sequences from sea anemones containing Kunitz-type peptides. (**a**) The conserved cysteine residues are highlighted with green text on yellow background. T, C, F, and M respectively represent tentacles, column, mesenterial filaments, and combine, highlighted in blue, orange, green, and yellow. (**b**) Homology modeling prediction of mature peptides sea anemone HC-50 and HC-47 with SHPI-1 (PDB 3M7Q).

**Figure 8:**
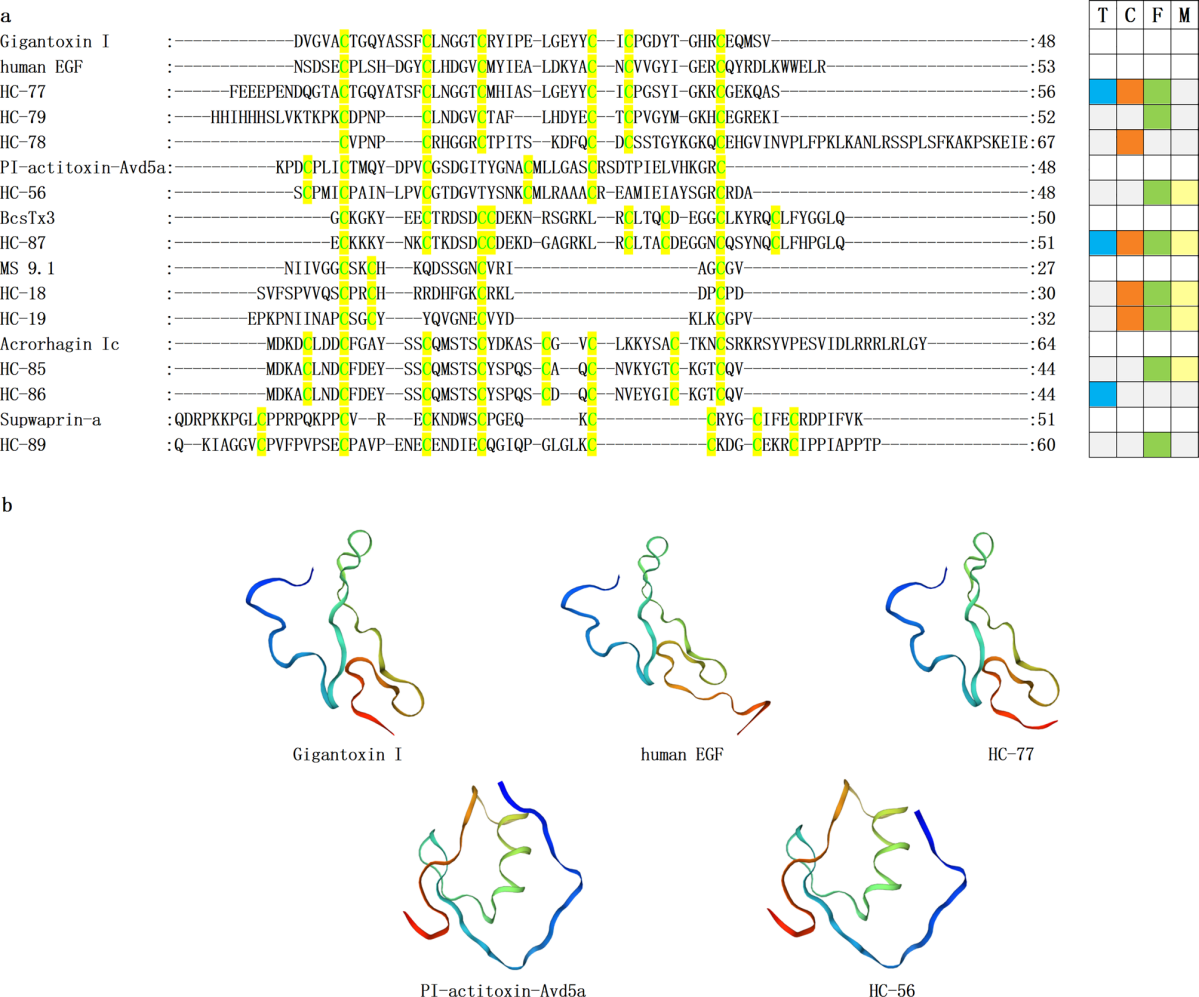
Representative Sea anemone mature peptide sequences in other families. (**a**) The conserved cysteine residues are highlighted with green text on yellow background. T, C, F, and M respectively represent tentacles, column, mesenterial filaments, and combine, highlighted in blue, orange, green, and yellow. (**b**) Homology modeling prediction of several representative sea anemone mature peptides with human EGF (PDB 7SZ1), PI-actitoxin-Avd5a (PDB 1Y1B).

Sea anemone venom has a high concentration of peptide toxins, serving a crucial function in prey and defense^7^, and they operate on various ion channels, such as Nav channels, Kv channels, ASIC, TRPV1, and TRPA1^29,43–45^. The three-dimensional (3D) structure or cysteine pattern or both of nine representative anemone peptide toxins have been determined, including ShKT domain, epidermal growth factor-like (EGF-like), β-defensin-like, Kunitz-type, *Anemonia sulcata* toxin III (ATX-III), inhibitor cystine-knot (ICK), small cysteine-rich peptides (SCRiPs), proline-hinged asymmetric β-hairpin (PHAB), and boundless β-hairpin (BBH)^41^. Herein, we described sea anemone peptide toxins with typical and unique homologs, including ShKT domain (11 homologs), β-defensin-like (11 homologs), Kunitz-type (7 homologs), and EGF-like (3 homologs). The peptide toxin sequences from these representative families were analysed to assess their similarity. The distribution of these sequences in these four datasets was examined. Additionally, the 3D structures of select significant peptide toxins were predicted (Figs 5, 6, 7 and 8).

The ShK toxin, isolated from the sea anemon *Stichodactyla helianthus*^46^, is termed the ShKT domain inhibits Kv channels^16,17,47–50^. A total of 11 homologous sequences with ShK that have not been reported previously were assessed, their cysteine pattern was C-C-C-CX3CX2C, and it predicted that the connection mode of disulfide bonds was C1-C6, C2-C4, C3-C5 (Fig. 5a,b). The sequence alignment demonstrated that HC-39 had the highest similarity with the previously identified sequence ID (GenBank No. XP_031556600.1), and the sequence identity was 85.71%. Additionally, the sequence similarity of HC-36/37/43 with ShK was 30.77%, 52.94%, and 30.30%, respectively. The homology modeling prediction indicated that HC-36/37/43 and ShK have 3D structure similarities. This suggests they may simultaneously act on Kv channels (Fig. 5c). These peptide sequences are all analogues of ShK and deserve further study.

β-defensins are ubiquitous in vertebrate antimicrobial peptides and are part of the main components of the innate immune system^51–53^. However, β-defensin-like peptides in sea anemone venom including CgNa, Rc I, Am II, BDS I, APETx1, APETx2, and Magnificamide are potential toxins that may disrupt voltage- and ligand-gated ion channels as Nav types 1/2/4, Kv type 3, ASIC, and ASIC3^54–60^. CgNa can be purified from the sea anemone *Condylactis gigantea* and inhibit Nav types 1/2^61^. Rc I is a peptide toxin in *H. crispa*, which can inhibit Nav channels^62^. Am II is a neurotoxin from *Antheopsis maculata* with toxin-paralyzing activity against crabs^63^. BDS I is a peptide toxin with an anti-angiogenic activity from the sea anemone *Anemonia viridis*^64^. APETx1 and APETx2 are peptide toxins with antibacterial and neurotoxic activity from *Anthopleura Elegantissima*. These toxins act on ERG Kv and Nav channels and ASIC3^56,65–68^. Magnificamide, a peptide inhibitor of mammalian α-amylases, isolated from the venom of sea anemone *Heteractis magnifica*, can be used to control postprandial hyperglycemia in diabetes mellitus^69^. Therefore, its functionally active recombinant analogue is a promising agent that awaits further investigation as a potential drug candidate for the treatment of type 2 diabetes mellitus^70^.

In this study, we identified 11 homologous sequences to sea anemone toxin β-defensin-like peptides with a cysteine pattern of CXC-C-C-CC (Fig. 6a,b). This compact core β-defensin structure relies on disulfide connections formed between cysteines C1-C5, C2-C4, and C3-C6. All these 11 novel homologous sequences were not previously reported. Sequence similarity analysis indicated that the sequence identity of HC-71 and Rc I (GenBank No. P0C5G5.1), HC-66/70 and Am II (GenBank No. P69930.1) was 97.87%, 97.83%, and 95.65%, respectively. Furthermore, HC-64/73 showed high similarities with previously reported sequences (GenBank No. ALL34531.1 and GenBank No. ALL34540.1), 93.33% and 80.00%, respectively. Taking CgNa (PDB 2H9X), BDS I (PDB 1BDS), and APETx2 (PDB 2MUB) as the templates, homology modeling prediction showed that HC-71 and Rc I, HC-64/68, and BDS I, HC-65/69 and APETx2 have similar 3D structures, respectively (Fig. 6c). Therefore, these peptide toxins may block multiple ion channels, including Nav, Kv, and ASIC.

Kunitz-type peptides are ubiquitous in marine organism venom^71,72^. Kunitz-type peptides block ion channels and are anti-inflammatory^73^. Kunitz-type peptides in sea anemones affect TRPV and type II Kv channels^74–76^. The ShPI-1 belongs to the Kunitz-type peptide, which is derived from the sea anemone *Stichodactyla helianthus*^77–79^. The ShPI-1 is toxin-suppressing Kv channels and proteases^78^. SHPI-1 is active against serine proteases, cysteine proteases, and aspartic proteases^80,81^. Seven homologous sequences with ShPI-1 were identified, and except for HC-52, the cysteine patterns of other sequences were C-C-C-C-CX3C, comprising three disulfide bridges with C1-C6, C3-C5, C2-C4 connectivity (Fig. 7a). Of the seven newly discovered sequences in Kunitz-type, HC-49 has the highest similarity of 84.75% to the reported sequence (XP_031567285.1). Furthermore, HC-47/50 and SHPI-1 have similar 3D structures, with a similarity of 56.36% and 54.72%, respectively (Fig. 7b). Therefore, HC-47 and HC-50 may have similar biological activities to SHPI-1.

EGF has a crucial role in the growth, proliferation, and differentiation of various cells of vertebrates^82,83^. The tight association between EGF-like peptides and the pathogenesis of human cancer is indicated by their diverse functionality in several cancer cell types, including bladder and liver cancer, as observed in mammalian EGF and its family members^84–87^. In invertebrates, particularly among hazardous marine creatures, the associated proteins have garnered considerable recognition due to their transformation into defensive and predatory toxins^88^. L-EGF is a growth factor released by the gastropod mollusk *Lymnaea stagnalis*^88,89^. The peptide toxin Gigantoxin I (ω-stichotoxin-Sgt1a) from *Stichodactyla gigantea*, acting on the Nav channel, can paralyze crabs^89,90^. Gigantoxin I is the first peptide toxin of the EGF family and is representative^88^. Here, we detected three homologous sequences with Gigantoxin I, and their cysteine patterns were C-C-C-CXC-C (Fig. 8a). The sequence identity of Gigantoxin I, HC-77, and Human EGF are 35.71% and 36.36%, respectively. Using Human EGF (BDP 7SZ1) as a model revealed similar 3D structures of Gigantoxin I and HC-77 (Fig. 8b). Therefore, invertebrate EGF family members, including three identified homologous sequences, may have similar biological activities to mammalian EGF.

Kazal-like belongs to serine protease inhibitor family and plays crucial roles in host physiological blood coagulation^91,92^, development regulation, and immunological functions^93^, in which protease activity is modulated by protease inhibitors^94^. PI-actitoxin-Avd5a is an elastase inhibitor from *Anemonia sulcata*, a 'non-classical' Kazal-type protein, and PI-actitoxin-Avd5a reveals strong inhibition against Streptomyces griseus protease B (SGPB)^95,96^. Taking PI-actitoxin-Avd5a (PDB 1Y1B) as a template for homology modeling, PI-actitoxin-Avd5a and HC-56 revealed similar 3D structures. Accordingly, HC-56 could strongly inhibit SGPB as PI-actitoxin-Avd5a.

ICK is a family of structural peptides that exerts its effects by targeting ion channels and serving as a defense mechanism against pathogens^97^. ICK is found abundantly in various species, and ICK toxins are also prevalent in animal venom that contribute to predation and defense^97,98^. BcsTx3, an ICK representative, is a Kv channel blocker from *Bunodosoma caissarum*. BcsTx3 mainly inhibits Kv channels, including but unlimited to Kv1.1, rKv1.2, hKv1.3, and rKv1.6. It also paralyzes swimming crabs when injected at the junction between the body and the walking leg^99^. Using blast alignment, the similarity between HC-87 and BcsTx3 (GenBank No.C0HJC4.1) sequences is as high as 72%. BcsTx3 and HC-87 have the same cysteine pattern (C-C-CC-C-C-C-C) (Fig. 8a). Therefore, it can be deduced that HC-87 may have one or all activities of BcsTx3.

MS 9.1, a positive modulator of mammalian TRPA1, is a typical representative of the BBH family^41^. TRPA1 is a non-selective cation channel involved in various physiological processes and exhibits significant anti-inflammatory and analgesic activities^15,100–102^. The homologous alignment results showed that HC-18 was the same as the sequence (GenBank No. BAS68532.1) from sea anemone Heteractis aurora. The similarity of HC-18/19 was 87.5%, and they share the same cysteine pattern. The HC-18/19 compounds that have been identified belong to the BBH family. It is hypothesized that the target of these compounds is TRPA1, providing a basis for developing drug screening assays aimed at identifying potential anti-inflammatory and analgesic medications.

Acrorhagin Ic obtained from red waratah sea anemone *Actinia tenebrosa* in New Zealand and Australian, is a member of the Acrorhagin family. HC-85/86 sequence is highly similar to the previously reported Acrorhagin Ic sequence (GenBank No.ATY39990.1), and the sequence identity is 61.90% and 59.52%, respectively. Although HC-85/86 and Acrorhagin Ic have similar 3D structures, Acrorhagin Ic is a toxin lethal to crabs inactively against any ion channels with no bacteriostatic activity, suggesting this peptide could have various biological functions^103,104^. Furthermore, HC-89 is also highly similar to the Supwaprin-a sequence (GenBank No.XP_048584021.1), a peptide from *Nematostella vectensis* (starlet sea anemone), and its sequence identity is 60.78%.

### Phylogenetic analysis of *H. crispa* sea anemone peptides

**Figure 9:**
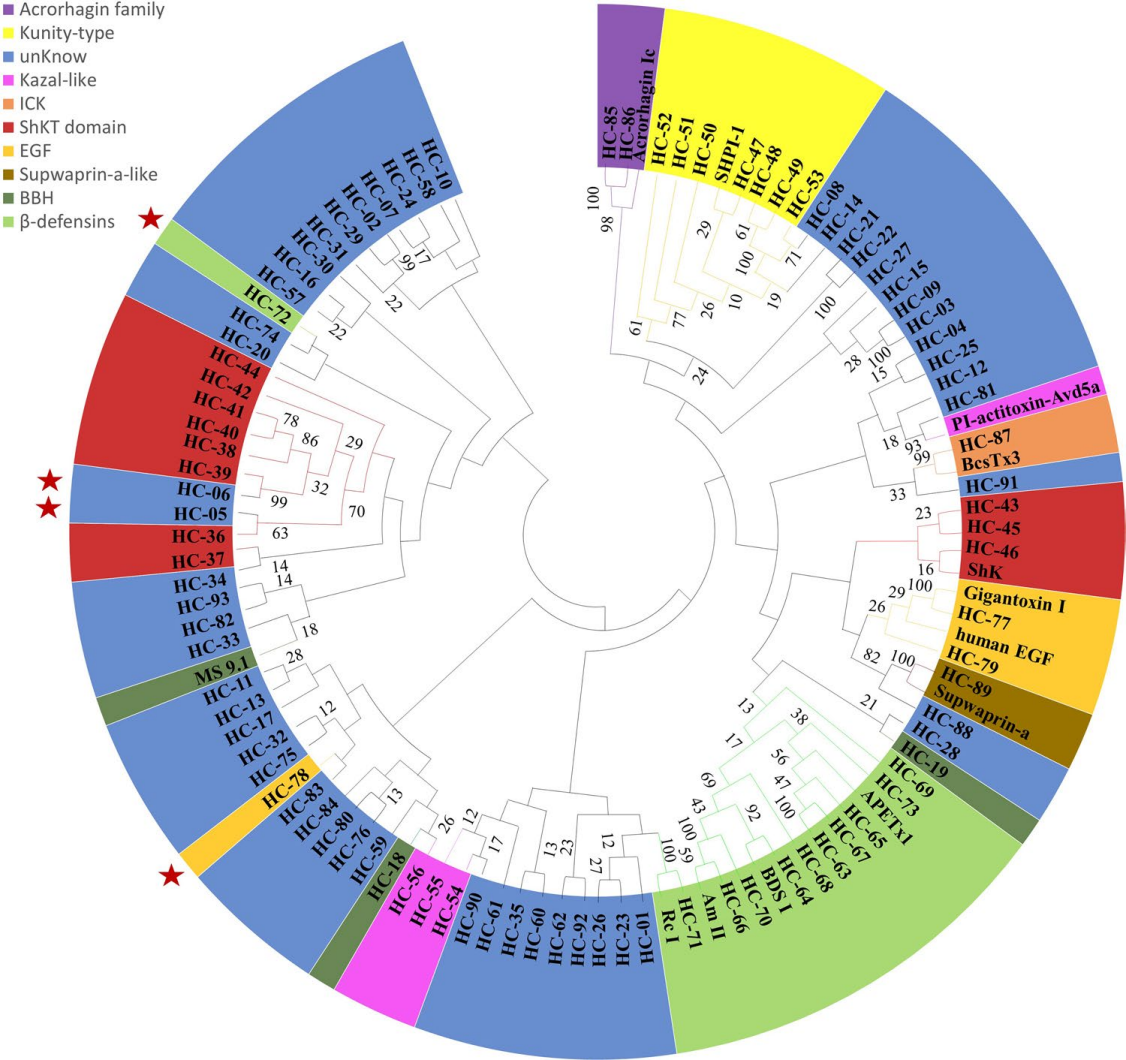
Phylogenetic tree from sequenced 93 peptide sequences and reported sequences in the Blast database. The tree was established by the NJ approach. Sequences with the same background color indicate peptides from the same family.

A total of 106 peptide sequences, including the mature regions of 93 peptide sequences from sea anemone *H. crispa* and 13 peptides of known families, were clustered using MEGA 7.0.14 software^105,106^. The results of phylogenetic analysis indicated that all peptides were divided into five major categories, some of which were consistent with the family classification based on the cysteine pattern, and there were also large differences in the family classification of a large part of peptides (Fig. 9). Among them, these β-defensin-like peptides (except for HC-72), Acrorhagin family, EGF (except for HC-78), and Kunitz-type family peptides can be clustered into one branch, respectively, showing good evolutionary and affinity relationship, consistent with the classification results based on cysteine pattern and structural characteristics. ShKT domain, Kazal-like, and BBH family peptides are clustered into two or more branches. This indicates their low similarity amino acid sequences, suggesting that there may be more diversity in target and activity. HC-05/06 were unknown families but were embedded in the ShKT domain. It is speculated that they are highly similar to the ShKT domain sequences with similar activities. A total of 52 unknown family peptides are distributed in nine major categories in the phylogenetic tree, indicating that family classification relies on amino acid similarity which provides more basis for the family classification of sea anemone peptide toxins.

## Discussion

HTS technology has been applied to multiple venom components of sea anemones^39^, including Exaiptasia diaphana, Nematostella vectensis, Oulactis sp., Anemonia sulcata, Megalactis griffithsi, Epiactis japonicus, Exaiptasia pallida, Anthopleura dowii, and Stichodactyla haddoni^30,32,33,40,107–113^. The HTS and bioinformatics techniques have been used to determine venom assemblage in tentacles, column, and mesenterial filaments for three species of sea anemone: H. crispa, Anemonia sulcata, and Megalactis griffithsi. A significant diversity was reported in the abundance of toxin-like genes across tissues and species^30^. However, specific sea anemone H. crispa crude venom components are unclear. Integrating HTS and bioinformatics technology can explore the peptide components in crude venom of sea anemone H. crispa, predict its family classification, and 3D structural and functional annotation.

Three sea anemone *H. crispa* tissues were sequenced and analyzed to explore peptide toxin distribution. Our HTS analysis detected 1049 transcripts, including 416, 291, and 307 putative protein and peptide toxin transcripts, respectively, from the tentacles, column, and mesenterial filaments. Whether these putative protein and peptide toxins are present in the venom needs to be further verified through technologies such as proteomics. Using the combined proteomic and transcriptomic techniques, a holistic overview of the venom arsenal of the well-studied sea anemone was obtained^32^. Macrander et al^30^, analyzed three sea anemone *H. crispa* tissues, including the tentacles, column, and filament, obtaining 840 protein and peptide toxins, and the toxin expression levels in the tentacles were significantly higher than those in the column and filament. However, we found that the protein and peptide families of the top 10 TPM sequences in the three tissues are similar, with the overall expression levels of all toxins being highest in the column of sea anemone *H. crispa*, followed by the tentacles and mesenterial filaments. Through comparative analysis, we found that there were significant differences between individuals even within the same species of sea anemone, and determining organizational boundaries may affect the data results. Among the 1049 protein and peptide sequences identified in this study, 88 sequences had a similarity of over 80% with the 840 sequences identified by Jason Macrander^30^. Among these 88 sequences, the main families of protein toxins are metalloproteinase and protein inhibitor, and the family of peptides is β-defensins and Kunitz-type (Figure S7a). However, a comparative analysis of 93 peptide sequences with previous study data revealed that only 22 sequences shared a similarity of over 80% (Figure S7b). Therefore, using our peptide screening principle, only 208 peptides were screened from 840 sequences identified by Jason Macrander^30^. Comparative analysis of cysteine in peptides showed that in previous studies, 142 peptides had cysteine residues less than 4, and only 66 peptides had cysteine residues greater than or equal to 4. These 93 peptide sequences all contain 4 or more cysteine residues, with the sequences containing 6 cysteine residues being the most abundant ones (Figure S7c). As is well known, most active sea anemone peptides such as β-defensins, ShK, Kunitz-type and other family toxins all contain 6 cysteine residues. In addition, comparative analysis was conducted with the traditional isolation of 48 protein and peptide sequences from sea anemone *H. crispa* and *H. magnifica*, and it was found that HC-71 and Rc I from *H. crispa* only differ by one amino acid, but there is no similar sequence from *H. magnifica,* indicating the diversity of sea anemone peptide toxins. Therefore, there are significant differences in toxin levels between individuals of the same species, especially anemones from different sea areas.

Herein, the proportion of identified proteins and peptide toxins is significantly different from previous studies. For example, the proportion of peptide sequences (9%) in sea anemone *Stichodactyla haddoni*^32^ and *Exaiptasia diaphana*^40^, was significantly lower than the proportion of protein sequences (91%) in our study. The relatively low proportion of peptides in the entire transcriptome of sea anemones can be attributed to the high concentration of protein in the column and mesenterial filaments. Most protein components corresponded to Peptidase S1, metalloprotease, G-protein coupled receptor, Factor 5/8 C-domain, and actinoporins. The actinoporins family, which holds significant recognition, has been identified for its possession of hemolytic action^28^. The sea anemone *H. crispa* actinoporin has in vitro anticancer activity, and it is expected to become an anticancer drug with high anti-migration potential^26,114^. In addition, the peptide constituents were shown to align with the ShKT domain, β-defensin, and Kunitz-type. In general, comparable findings were noted in transcriptome data obtained from various sea anemones^32,33,40^. The protein component metalloproteinases were highly expressed in the tentacles and the mesenterial filaments, with lower expression levels in the column than in ShKT domain. Metalloproteinases significantly participate in wound-healing processes in cnidarians, including tentacles regeneration and transdifferentiating^115^, they also have potential dual involvement in food digestion development^116^. For peptide toxin components, the ShKT domain was highest expression in the column tissue of sea anemone *H. crispa*, followed by expression in the tentacles and a small amount in the mesenterial filaments. This also verifies that sea anemones do not have a centralized venom system, and the toxin peptide is expressed throughout the sea anemone, not just in the tentacles^30,117^. Surprisingly, β-defensin-like peptides are only highly expressed in the column, not in the other two tissues. β-defensin-like peptides, as antimicrobial peptides, can accelerate wound healing, are widely present in vertebrates, and are one of the main components of the innate immune system^51–53,118^. β-defensin peptides showed paralytic activity in crustaceans, indicating that it had evolved into a weapon to capture prey^119^. Here, β-defensin-like peptides are not highly expressed in the tentacles and may have other biological functions.

The most common and pharmacologically valuable peptides in sea anemones are the ShKT domain, β-defensin-like, Kunitz-type, and EGF-like peptides, and they influence various ion channels, including Nav channels, Kv channels, ASIC, TRPV1, and TRPA1^29,43–45^. The ShKT domain is one of the families with the highest presence in the transcriptome data of sea anemone *H. crispa*, suggesting that this type of peptide toxin may play a crucial role in its predation, defense, and competition^17,47,48^. ShK inhibits Kv channels, blocking Kv1.1/1.2/1.3/1.6/3.2 and Kca3.1 channels, especially in Kv1.1/KCNA1 and Kv1.3/KCNA3 channels^16,49,50^. Kv1.3 is involved in various autoimmune diseases and many cancers by contributing to cell proliferation, malignant angiogenesis, and metastasis^18,20,120–125^. ShK is a Kv1.3 channel blocker analogue with significant roles in T and B lymphocyte subsets related to autoimmune conditions. Therefore, ShK is a potential immune modulator for autoimmune disease therapy^46^. Of these, ShK-186, also known as Dalazatide, was the first representative of the ShKT domain to be detected and characterized and the first drug to complete Phase I trials^17,46,126^. ShK and its analogues, including 11 homologs in the ShKT domain found in this study, may act on Kv1.3, suggesting that they may have significant involvements in treating human autoimmune disorders^127–129^.

β-defensin-like peptides block ligated-gated and voltage-gated ion channels, as Nav types 1/2/4, Kv type 3, and ASIC^54–57,130^. Eleven identified β-defensin-like homologous sequences may act on Nav types 1/2/4 channels related to acute and chronic pain, and it can potentially treat pain^131,132^. Additionally, these peptides acting on Nav channels, considered insecticidal lead compounds, have insecticidal effects^133^. Kunitz-type peptides block ion channels and are anti-inflammatory^73^. HCRG1/2 are the first Kunitz-type peptides to block Kv1.3 found in sea anemones^27,134^. The first Kunitz-type representative bovine pancreatic trypsin inhibitor (BPTI) is a serine protease inhibitor resisting inflammatory responses^135,136^. In sea anemones, Kunitz-type peptides act on TRPV1 and Kv channels^29,74,75^, indirect TRPV1 activation contributes to EGF receptor/PLA2/arachidonic acid/lipoxygenase pathway, resulting in Kunitz-type peptides regulating TRPV1 channel activity^41,137^. APHC1-3 is earlier shown to possess a unique property of inhibiting of the pain vanilloid receptor TRPV1 in vitro and providing the analgesic effects in vivo in addition to their trypsin inhibitory activity^76^. The activated ion channel TRPV1 produces pain, so TRPV1 is the most important therapeutic target for pain and inflammatory stimulation^14,29,75,138^. Besides blocking TRPV1 channels, various anemone Kv channel toxins inhibit serine protease activity, participating in various functions, like blood clotting, tumor immunity, fibrinolysis, inflammatory modulation, and resistance against bacterial and fungal infections^73,139,140^. Seven homologous Kunitz-type peptide sequences were identified, contributing to the anti-inflammatory responses by inhibiting serine protease activity and Kv channels.

The phylogenetic tree of typical family sea anemone peptides exhibited a pattern in which the majority of the sequences were clustered based on families, while a subset of individual sequences remained dispersed among alternative family groupings. The peptide sequence families of the transcriptomes in this study were based on changes in cysteine patterns and 3D structures, resulting in some sequences not being clustered together. Therefore, 3D structural alignment is a very powerful tool for inferring the evolutionary relationship between two low homology peptides.

## Conclusions

The transcriptome analysis of *H. crispa* sea anemone venom from the tentacles, column, and mesenterial filaments was performed using HTS technology. A total of 1049 putative protein toxins were obtained, including 956 (91.0%) protein sequences and 93 (9.0%) peptide toxin sequences, which were divided into 60 known families. ShKT domain in peptide toxins was predominantly expressed in the tentacles, column, and mesenterial filaments and contributes to prey capture, defines, and intraspecific competition. Our study demonstrated that the venom assemblages within these different sea anemone *H. crispa* tissues are complex and diverse. Combining HTS and bioinformatics technologies new peptides can be systematically identified in addition to predicting their family categorization, 3D structures, and functional annotations. These advances lay the foundation for enhanced understanding and development of sea anemone venom as potential marine pharmaceuticals.

## Materials and methods

### Specimens and RNA extraction

The sea anemone was collected from Paracel Islands located at [Lat 15°46' N, Lon 111°11' E] in the Southern Sea of China and maintained in the lab in aquariums containing artificial seawater. The sea anemone sample was identified by the mitochondrial genome as *H. crispa*. A total of three *H. crispa* were collected, and over a week, different tissues were removed from *H. crispa* using tweezers and a scalpel, starting with the tentacles, column, and mesenterial filaments including the pharynx and gonads. Three different *H.crispa* tissue samples were mixed separately, and then the total RNA from these three tissues were extracted after liquid nitrogen flash evaporation (TIANGEN biotech Co., Ltd., China), and their RNA integrity number values were measured using an Agilent 2100 Bioanalyzer (Agilent Tech., Palo Alto, CA, USA). Then, BGI-Tech (Shenzhen, Guangdong, China) was used to build three Illumina cDNA libraries from qualifying RNAs and sequenced them using an Illumina HiSeq4000 platform (San Diego, CA, USA).

### Sequence analyses and assembly

This study evaluated the assembly integrity of four assembled transcripts using BUSCO v5.2.2 software and databases: etazoan_ Odb10 (Creation date: 2021-02-17, genomes: 65, number of BUSCOs: 954). BUSCO was run in mode: transcriptome. Illumina HTS, raw image data, was converted into raw reads after base calling by Illumina CASAVA software (v1.8.4). High-quality clean reads were obtained by removing the adapter and reads with > 10% of non-sequenced bases or > 50% of low-quality bases (≤ 10 was the base quality score). We compared the transcriptomes after assembly to evaluate the impact of the cleanup step on overall completeness and also conducted a reciprocal BLAST (Basic Local Alignment Search Tool) search of known sea anemone venom genes to determine whether the alternative cleanup strategies would result in a different number of candidate toxin genes. The transcriptome sequence assembly strategy was used to assemble HTS data into transcript sequences through three steps of Inchworm, Chrysalis, and Butterfly^36,37^. (A) Inchworm: Use Kmer-based assembly strategy to assemble reads into contigs, (B) Chrysalis: Cluster contigs sequences, define components, and align reads back to components to verify correctness, (C) Butterfly: De Bruijn graph-based assembly strategy to assemble components into possible transcripts. This study generated four transcriptome reference sequences for Tentacles, Column, Mesenterial filaments, and their combination (Combine).

### Gene annotation

The Unigene gene's coding region was predicted using the translation approach, and possible coding protein sequences were predicted. The resulting protein sequences were cross-referenced against Uniprot and the non-redundant (NR) protein database (https://blast.ncbi.nlm.nih.gov/Blast.cgi). The protein coding model was determined by utilizing the coding mode of the alignment with the highest alignment score. Unigene gene annotation was conducted based on NR, Uniprot, KEGG^141–143^, and KOG (eukaryotic)/COG (prokaryotic) databases.

### Cluster heatmap and GO analysis

Differentially expressed genes (DEGs) between two tissues were determined by a Fold Change (FC) of | LogFC |>2. The gene expression heatmap is plotted using the TPM value and the R language's photomap package (Pretty Heatmaps (Version 1.0.12) method. GO enrichment analysis of DEGs was conducted by using the cluster profiler program^144^. Fisher's exact test pvalue or Benjamin’s corrected pvalue less than 0.05 was set as the significant enrichment level.

### Identification of protein and peptide toxins

Prediction of sea anemone protein and peptide toxins using four datasets, homolog searches, and an ab initio prediction method (tentacles, column, and mesenterial filaments, Combine). The BLAST database was queried for proteins and peptides for sequence similarity prediction. After assembly, the sequences were checked against a local database using BLASTX (with an E-value of 1e-5). The BLASTX-hit unigenes were used to generate amino acid sequences. According to the BLAST database's superfamily and family classifications, those four datasets were divided into different groups.

Prediction and comparison of 183,198 transcripts of *H. crispa* were completed by using SPM Predictor (length ≤ 200, hydrophobic ≥ 70%), Diamond ATDB database (with an E-value ≤ 1e-8) and Diamond NR database (with an E-value ≤ 1e-8). A Python script was developed to trim all of the sea anemone toxin-candidate transcripts to allow only the open-reading frame (ORF) identified by Transdecoder (http://transdecoder.github.io).

### Classification of protein and peptide toxins superfamilies

Using the BLAST (default setting), predicted sea anemone peptide and protein transcripts were identified. Peptide and protein toxins with the highest resemblance to known superfamilies in the BLAST database were assigned based on cysteine structural scaffold. Those protein and peptide toxins with low similarities (< 75%) were classified into unknown groups.

### Alignment and homology modelling

MEGA 7.0.14 software was used to create new protein sequence alignments and perform amino acid alignments on all peptide sequences, where the MUSCLE algorithm was chosen to intelligently align amino acids^105,106^. Genedoc software was used to export the sequence in FASTA format.

Protein 3D structure was predicted using homology computational structure prediction modeling from amino acid sequences^145^. The SWISS-MODEL, available through the Expasy web server or Deep View software (Swiss Pdb-Viewer) were applied. The homologous sequences with high sequence identity were assigned as templates, and then the cartoon mode was used to build the model.

### Phylogenetic analyses

Representative peptide sequences from various families of sea anemones were obtained from UniProt and BLAST databases (www.uniprot.org/, https://blast.ncbi.nlm.nih.gov/), and they were comparable to those sequences obtained in this study. The mature regions of 93 peptide sequences were aligned using MEGA 7.0.14. A phylogenetic tree was established using the Neighbor-Joining approach (Bootstrap method 1000 and Pairwise deletion 50%).

### Human and animal resources

The article does not involve human or animal experiments, and all sea anemones are collected according to the collection permit issued by the China Fisheries Administration.

### Supplementary Information

Supplementary Figures.

Supplementary Table S1.

Supplementary Table S2.

Supplementary Table S3.

Supplementary Table S4.

## Data Availability

The datasets described are accessible through internet repositories. The repository(s) and accession number(s) can be accessed at the following URL: https://www.ncbi.nlm.nih.gov/sra/PRJNA893400 (accession: SRX17999840, SRX17999841, and SRX17999842).
