# Supplementary material for: Diversity analysis of sea anemone peptide toxins in different tissues of Heteractis crispa based on transcriptomics
Source: Sci Rep. 2024 Apr 1;14:7684. doi: 10.1038/s41598-024-58402-2 (PMC10985097; doi:10.1038/s41598-024-58402-2)
Supplement: Supplementary file 1 — Supplementary Figures. [file 41598_2024_58402_MOESM1_ESM.pdf]

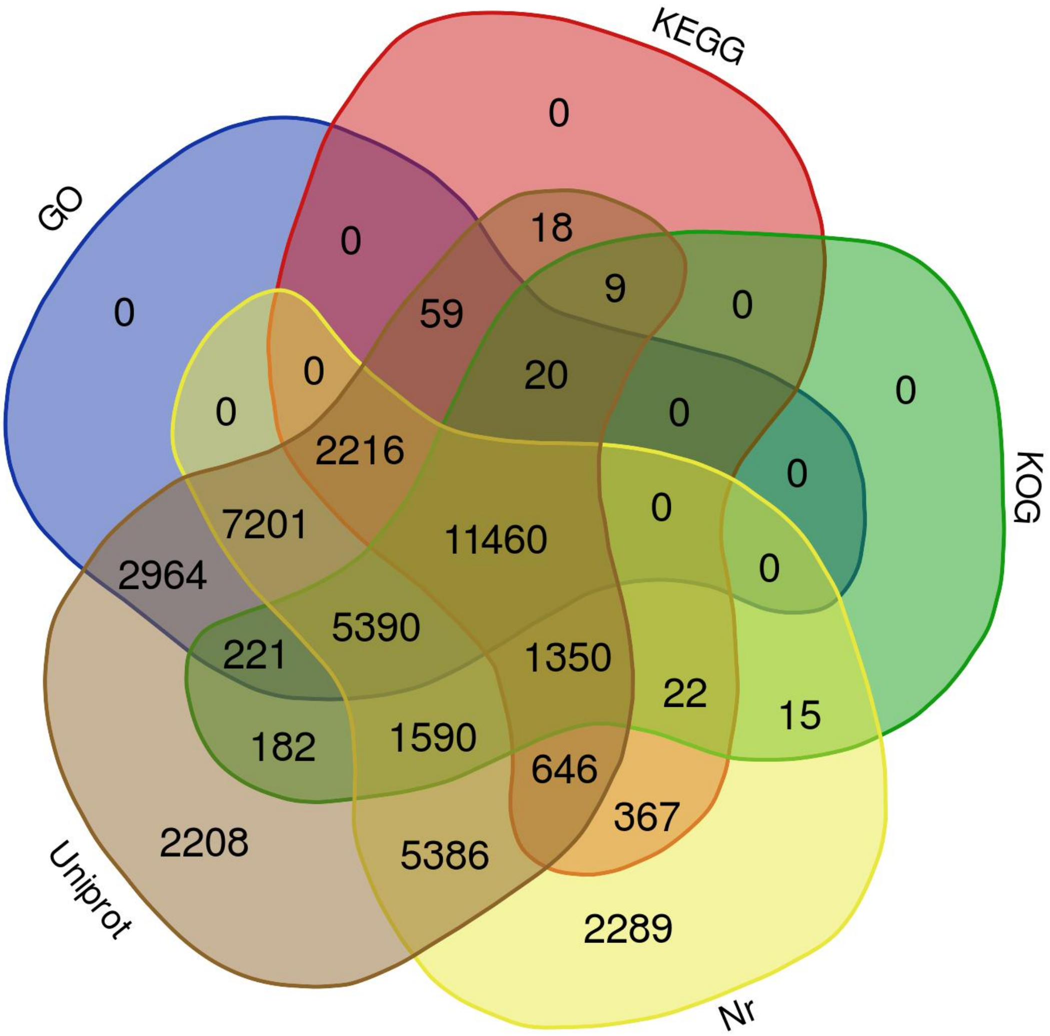

Supplementary Fig. 1 Function annotation on five databases, Nr, Uniprot, GO, KEGG, and KOG

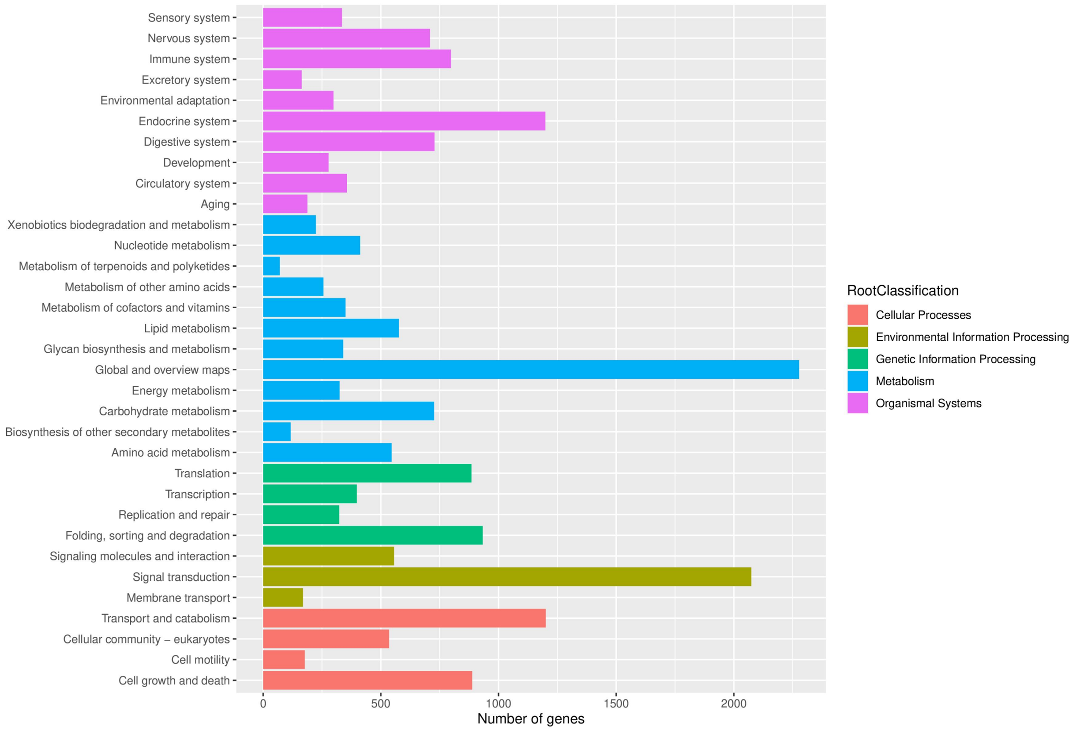

Supplementary Fig. 2 The distribution of annotated unigenes

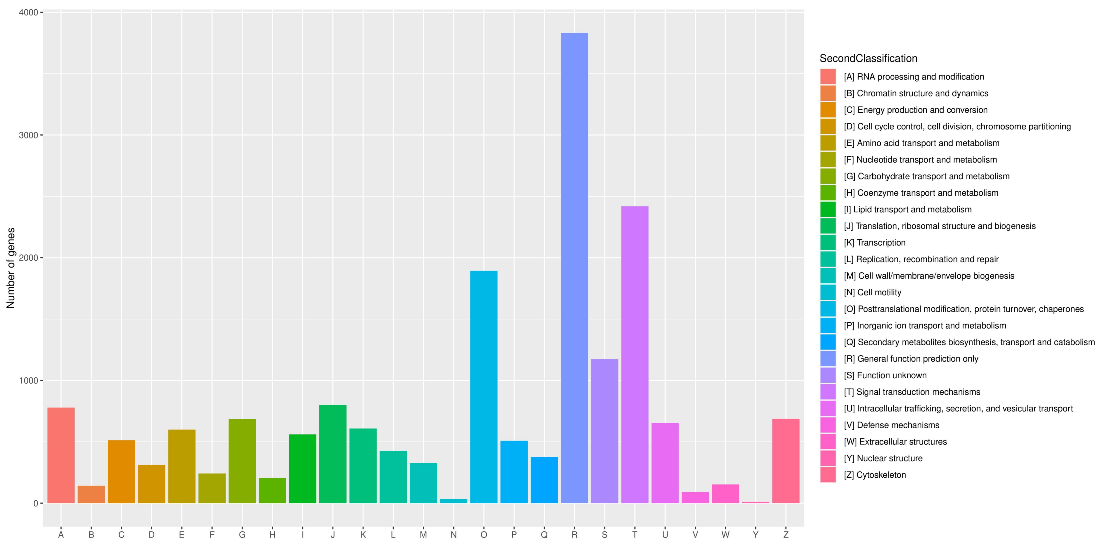

Supplementary Fig. 3 Predict and classify functions on the KOG database

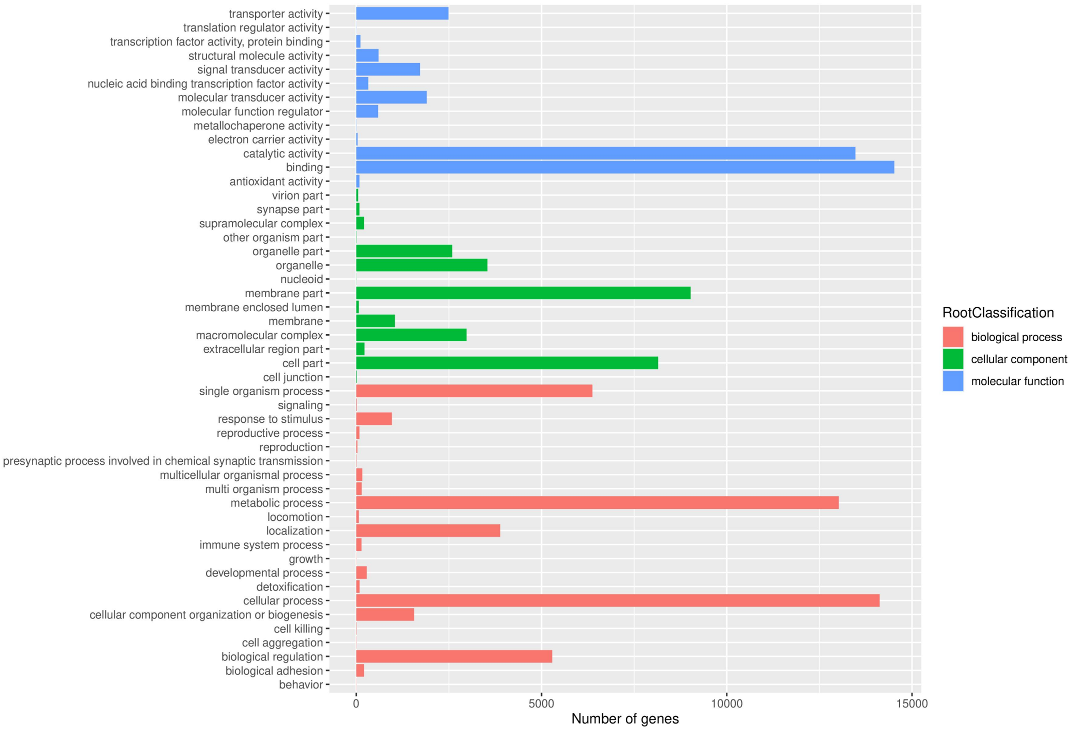

Supplementary Fig. 4 Annotated under three headings biological process, cellular component, and molecular function.

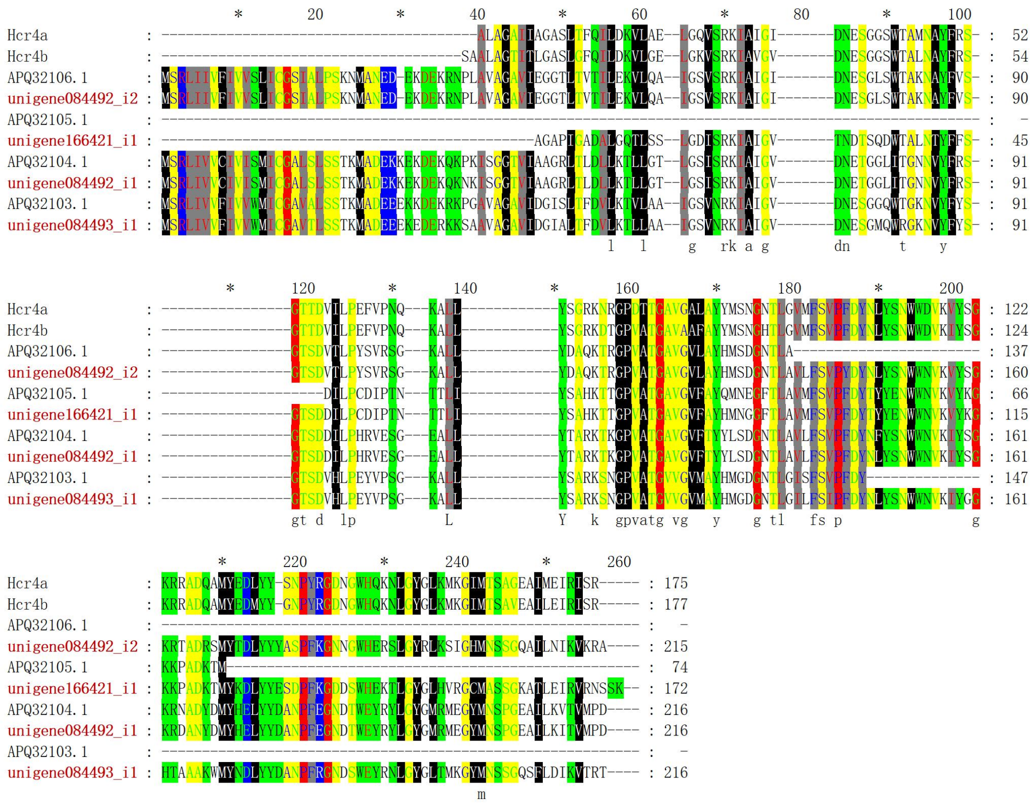

Supplementary Fig. 5 Sequence alignment between the actinoporins toxins sequence

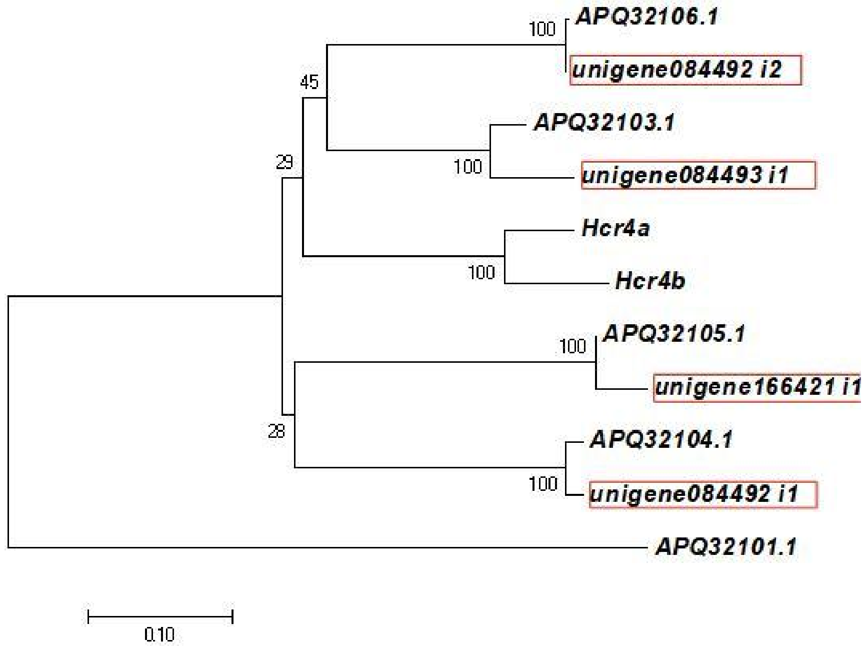

Supplementary Fig. 6 Phylogenetic tree of the actinoporins toxins sequence

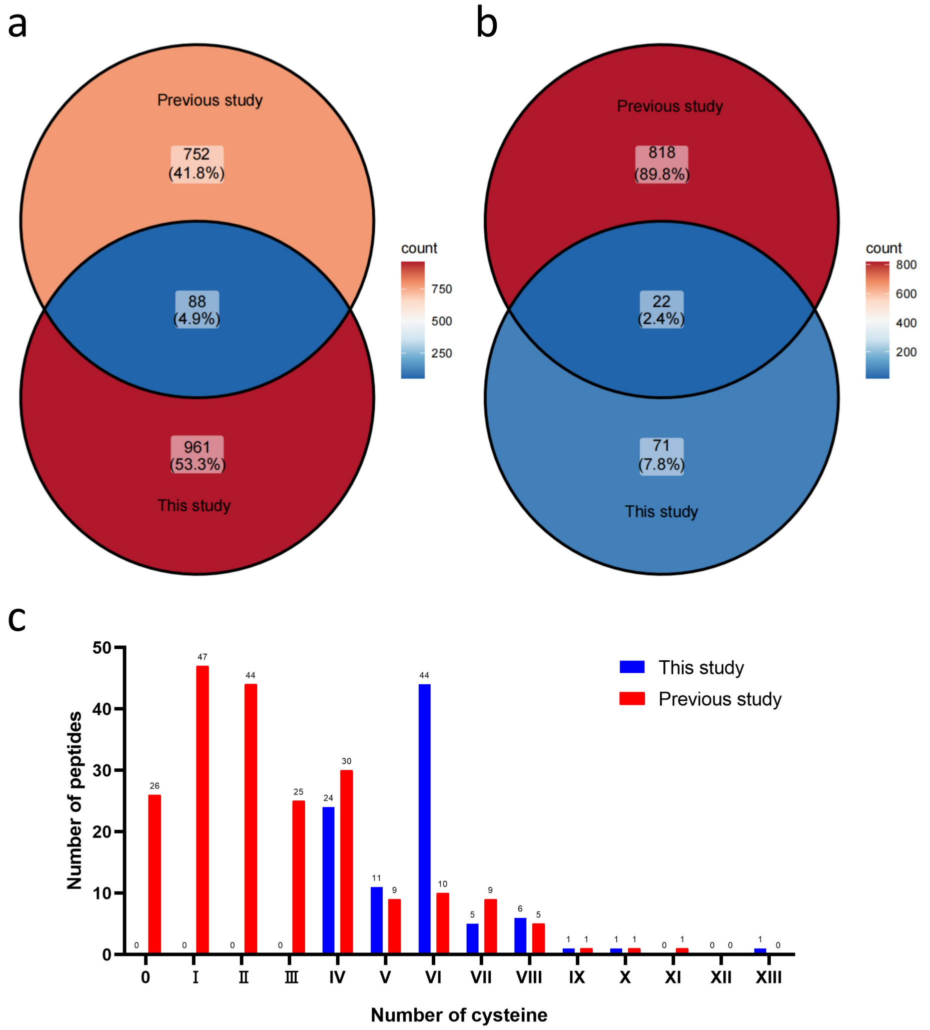

Supplementary Fig.7 Comparative analysis between the data from this study and those from previous study
