# Supplementary material for: Diversity analysis of sea anemone peptide toxins in different tissues of Heteractis crispa based on transcriptomics
Source: Sci Rep. 2024 Apr 1;14:7684. doi: 10.1038/s41598-024-58402-2 (PMC10985097; doi:10.1038/s41598-024-58402-2)
Supplement: Supplementary file 3 — Supplementary Table S2. [file 41598_2024_58402_MOESM3_ESM.docx]

**Supplementary Table 2.** *H. crispa* sequence statistics and assembly summary.

| **Samples** | **Combine** | **Tentacles** | **Column** | **Mesenterial filaments** |
| --- | --- | --- | --- | --- |
|  | **Raw data** | | | |
| **Total Reads** |  | 83,799,076 | 69,427,990 | 67,486,092 |
| **Total length(bp)** |  | 12,569,861,400 | 10,414,198,500 | 10,122,913,800 |
| **Read length(bp)** |  | 150 | 150 | 150 |
|  | **Clean data** | | | |
| **Total Reads** | 120,000,000 | 81,961,116 | 67,828,874 | 66235446 |
| **Total length(bp)** | 18,000,000,000 | 12,294,167,400 | 10,174,331,100 | 9,935,316,900 |
| **Read length(bp)** | 150 | 150 | 150 | 150 |
| **Clean data ratio** |  | 97.81% | 97.70% | 98.15% |
|  | **Contig** | | | |
| **Total Number** | 288,563 | 266,439 | 185,255 | 182,032 |
| **Total length(bp)** | 288,870,553 | 279,995,881 | 175,508,612 | 160,770,860 |
| **Mean Length(bp)** | 1,001 | 1,050 | 947 | 883 |
| **N50(bp)** | 1,829 | 1,901 | 1,863 | 1,700 |
| **N70(bp)** | 938 | 1,027 | 918 | 793 |
| **N90(bp)** | 376 | 395 | 343 | 323 |
| **GC content** | 43.18 | 43.92 | 40.70 | 41.59 |
|  | **Unigene** | | | |
| **Total Number** | 183,198 | 167,413 | 111,229 | 118,387 |
| **Total length(bp)** | 140,958,722 | 138,158,885 | 77,747,597 | 77,077,483 |
| **Mean Length(bp)** | 769 | 825 | 698 | 651 |
| **N50(bp)** | 1,199 | 1,367 | 1,202 | 991 |
| **N70(bp)** | 613 | 704 | 509 | 457 |
| **N90(bp)** | 312 | 324 | 273 | 267 |
| **GC content** | 44.97 | 45.80 | 40.80 | 42.21 |
